# Role of menopausal hormone therapy in the prevention of postmenopausal osteoporosis

**DOI:** 10.1515/biol-2022-0759

**Published:** 2023-12-12

**Authors:** Zhao Na, Wei Wei, Yingfang Xu, Dong Li, Beili Yin, Weiqun Gu

**Affiliations:** Department of Gynecology, Changshu Hospital Affiliated to Nanjing University of Chinese Medicine, Changshu, 215500, China; Department of Orthopaedics, Changshu Hospital Affiliated to Soochow University, First People’s Hospital of Changshu City, Changshu, 215500, China; Department of Obstetrics and Gynecology, Changzhou Geriatric Hospital Affiliated to Soochow University, Changzhou No. 7 People’s Hospital, Changzhou, 213000, China

**Keywords:** osteoporosis, menopausal hormone therapy, bone health, postmenopause

## Abstract

The use of menopausal hormone therapy (MHT) has declined due to concerns about its potential side effects. However, its pivotal role in managing postmenopausal osteoporosis is gaining increased recognition. In this article, we explore how MHT assists postmenopausal women in maintaining bone health and preventing fractures. Recent research indicates that MHT significantly reduces the risk of fractures in women. This benefit is evident regardless of a woman’s bone mineral density or their use of progestogens. However, there is limited evidence suggesting that the skeletal benefits continue once the treatment is discontinued. Possible complications of MHT include heart attacks, clots, strokes, dementia, and breast cancer. The most suitable candidates for MHT are women who have recently entered menopause, are experiencing menopausal symptoms, and are below 60 years of age with a minimal baseline risk of adverse events. The treatment is available to those who meet these criteria. For women undergoing premature menopause, MHT can be considered as a means to protect bone health, especially if initiated before menopause or if accelerated bone loss is documented soon after menopause. Such decisions should be made after evaluating individual risk factors and benefits.

## 1 Introduction

Over the past 30 years, osteoporosis has transformed from being perceived as a normal part of aging to being recognized as a significant non-communicable chronic illness. It now has clearly defined diagnostic criteria and effective means for early detection, risk assessment, and treatment. Osteoporosis is a skeletal disorder marked by decreased bone strength, which heightens the risk of fractures. This stems from a decline in bone density and a deterioration of bone microarchitecture. Fortunately, an array of treatment strategies aimed at increasing or preserving bone mineral density (BMD) are now accessible for osteoporosis management [1–3]. Oral bisphosphonate medication is frequently recommended for patients with diagnosed osteoporosis and a heightened fracture risk [1–3]. However, other pharmaceutical interventions such as zoledronic acid, denosumab, and teriparatide are tailored to specific stages of the risk spectrum. Identifying women who currently have a low risk but a high lifetime potential for fractures is crucial in the effective management of osteoporosis.

**Figure 1:**
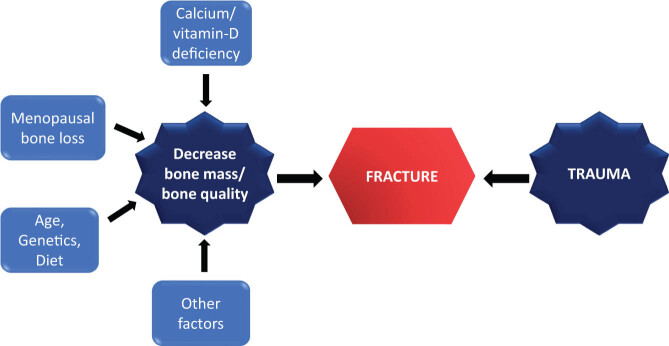
Physiology behind fractures caused by osteoporosis.

Inadequate bone mass or failure to achieve peak bone density can result in osteoporosis later in life (Figure 1). The attainment of optimal peak bone mass is influenced by both genetic factors and lifestyle choices, including nutrition, exercise, and abstention from bone-toxic drugs. Achieving this peak is vital in preventing osteoporosis. Factors such as poor nutrition during developmental years, lack of physical activity, coexisting medical conditions (e.g., diabetes mellitus, thyrotoxicosis, or Cushing’s syndrome), and certain medications (like corticosteroids or anticonvulsants) can contribute to a reduced peak bone mass. This peak typically develops during childhood, adolescence, and early adulthood [4]. Given its proven efficacy in fracture prevention, regardless of baseline bone density, and its potential benefits in alleviating menopausal symptoms, menopausal hormone therapy (MHT) stands out as a significant pharmaceutical intervention for osteoporosis management [5].

Hormone replacement therapy (HRT) was frequently prescribed during the 1980s and 1990s to treat menopausal symptoms such as night sweats, hot flashes, and sleep disturbances. Additionally, it was believed to confer benefits like osteoporosis prevention [6–8]. This strategy’s justification stemmed from observational studies correlating HRT with improved bone health. However, the Women’s Health Initiative (WHI) hormone therapy trials introduced skepticism. The first of these studies, published in 2002, determined that an HRT regimen combining conjugated equine estrogens (CEEs) with medroxyprogesterone acetate (MPA) did decrease fracture risk, but it also elevated the risk of cardiovascular and cerebrovascular events, breast cancer, and other health complications [9].

The evolving story of MHT serves as a cautionary tale, emphasizing the limitations of observational research and the potential for conflicting results when transitioning to randomized controlled trials [9]. Notably, re-analyses of the original observational studies using advanced pharmacoepidemiological methods – better accounting for confounding by indication – showed results more aligned with those from randomized trials [10]. Despite this, observational study outcomes inherently lack the reliability of those from randomized trials.

Further, different MHT protocols (considering the inclusion or exclusion of progestogen, the type and dosage of both estrogen and progestogen, and administration route) might lead to varying benefit–risk profiles. This notion is supported by additional re-analyses of the WHI and other studies [11]. The intent of this article is to provide a structured perspective on MHT, detailing the evidence supporting its use in preventing osteoporosis and related fractures.

## 2 Physiological aspects of menopause and its connection to bone

### 2.1 Menopausal physiology

The reduction of ovarian follicular activity leads to menopause, marking the permanent cessation of menstrual cycles. Typically, menopause is diagnosed in women in their forties or fifties, following a 12-month period of amenorrhea with no underlying physiological or pathological cause. The perimenopausal period, which usually spans 4–8 years, involves various endocrine, biochemical, and clinical changes that prepare the body for menopause [12,13].

During a regular menstrual cycle, the pituitary gland secretes luteinizing hormone (LH) and follicle-stimulating hormone (FSH), regulating the ovary’s production of estrogen, testosterone, and progesterone. However, due to ovarian reserve depletion and decreasing estrogen levels, menstrual periods may become shorter or irregular during perimenopause, even when estradiol levels remain relatively stable. When estrogen levels decline, FSH and LH levels rise in the bloodstream. Unlike estradiol, which decreases rapidly following menopause, testosterone, androstenedione, and dehydroepiandrosterone sulfate may gradually decrease with age. Postmenopausal women typically have low progesterone levels and reduced circulating estrogen levels, although local aromatization of androgen precursors may still allow for some estrogenic actions at the molecular level [10,14]. Menopausal symptoms, including hot flashes, sleep disturbances, mood swings, and urogenital changes, are attributed to these hormonal shifts and have been extensively discussed elsewhere [10].

### 2.2 Menopause and its relation to the skeleton

Albright [15] reported his findings linking estrogen insufficiency due to menopause to poor bone health, establishing the idea that exogenous estrogen therapy can assist in maintaining bone mass and reducing fracture risk. Estrogen functions as an antiresorptive drug by regulating the receptor activator of nuclear factor kappa B ligand (RANKL) pathway, thus reducing osteoclast numbers [16]. The absence of estrogen diminishes matrix production, but its treatment may rejuvenate it [17].

The menopausal transition is associated with accelerated bone loss, particularly in the trabecular bone, and an increased risk of fractures later in life. Menopausal hormone therapy (MHT) has proven effective for both menopause and spinal osteoporosis, as these two conditions are interconnected. Hip and vertebral fractures become significantly more prevalent at older ages, while wrist fractures become more common around the time of menopause [10,18]. Recent menopausal women who experience a wrist fracture should undergo a comprehensive risk assessment and take necessary precautions to minimize the likelihood of further fractures [10].

### 2.3 A brief overview of the history of MHT as well as its various types (estrogens and progestogens)

A brief overview of MHT reveals that it involves the administration of both estrogen and progestogen to mitigate the endometrial stimulation caused by estrogen. Hysterectomy patients only take estrogen replacement therapy. In the 1890s, climacteric symptoms were first treated with desiccated ovarian estrogen. In 1929, oestrone was extracted from the urine of pregnant women [19]. Initially termed “theelin,” it was later renamed “oestrin.” By 1936, oestradiol was derived from pig ovaries [10,20]. Progynon, the first estrogenic product on the market, was an injectable hormone originally obtained from the placentas of pregnant animals. In 1941, Canada began offering oral estrogen treatment using conjugated estrogens collected from the urine of pregnant mares [20–22]. In 1942, the US joined in the medical advancements. The first isolation of crystalline progesterone occurred in 1934 [20]. It was Russell Marker who pioneered its synthesis by extracting it from diosgenin in cabeza de negro, also known as Mexican yam. Initially, there was no debate regarding the use of estrogen treatment for women. However, by the mid-1970s, it was found that using estrogen alone heightened the risk of endometrial cancer, making the addition of a progestogen essential for endometrial safety. In industrialized countries, the usage of MHT has decreased since its peak in the 1990s, when researchers first began to associate oral conjugated estrogens with MPA and an increased risk of breast cancer [20]. There have been several publications in the past 20 years that explore the various MHT formulations and routes of administration, as well as meta-analyses of the WHI conjugated estrogens and MPA research and the conjugated estrogen-alone study data [17,22–25]. The following discussion of the perks and drawbacks of MHT is made possible by the insights gained from these studies, which in turn have improved clinical decision-making.

**Figure 2:**
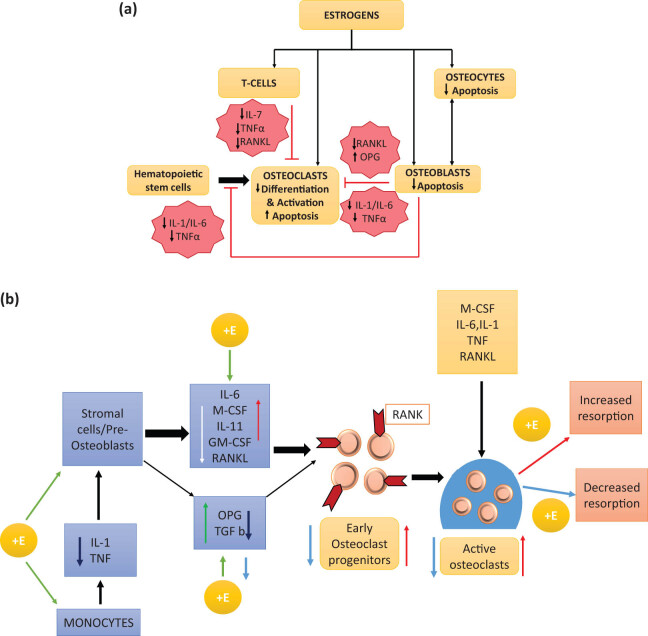
(a) Influence that estrogens have on bone cells. (b) Alterations in cellular state brought on by estrogen fluctuations. Effects are shown with +E when estrogen is present, while effects are shown with −E when estrogen is not present. IL-1 is also known as TNF and OPG. Estrogen inhibits osteoclastogenesis and enhances osteoclast apoptosis. Estrogen inhibits osteoclastogenesis by blocking the production of IL-1 and TNF. It also increases the susceptibility of stromal cells and preosteoblasts to the effects of IL-1, which in turn blocks the production of M-CSF, RANKL, and possibly most significantly, IL-6. Furthermore, estrogen encourages the formation of OPG, which is a powerful inhibitor of osteoclastogenesis. Additionally, estrogen has the effect of making osteoclast precursors less sensitive to RANKL. Additionally, estrogen encourages osteoclastic apoptosis, which in turn shortens the lifetime of osteoclasts. It would indicate that TGF-b is the factor responsible for causing this impact [4].

Inadequate estrogen levels during the early stages of the menopausal transition significantly contribute to alterations in bone mass and quality, playing a substantial role in the development of postmenopausal osteoporosis. Estrogen is crucial for both bone development and maintenance, as bone is an estrogen-dependent tissue. Estrogens act on osteoblasts through specific receptors (ER) and, by extension, influence osteoclast progenitors and T-lymphocytes, leading to a substantial reduction in osteoclast growth and activity (Figure 2a). Additionally, estrogens inhibit apoptosis (cell death) in osteoblasts and osteocytes, ensuring continuous bone synthesis [26]. Estrogen deficiency during menopause primarily results in increased bone resorption and cellular-level changes, with bone formation unable to compensate for the heightened resorption activity. Rapid bone loss and alterations in microarchitecture, such as disorganization, thinning, and rupture of bone trabeculae, are associated with an elevated risk of fractures, even when bone remodeling remains robust [27,28].

Estrogen deficiency during menopause results in an increase in osteoclastic resorption activity while osteoblastic activity remains relatively constant, ultimately leading to a net loss of bone. This phenomenon was originally termed “uncoupling.” Understanding the molecular changes that occur due to estrogen deprivation (Figure 2b) has been a research priority. Cells of the stromal/osteoblastic lineage become more sensitive to interleukin-1 (IL-1) and tumor necrosis factor (TNF)-β in response to estrogen deprivation. These cells, when exposed to IL-1 and TNF, secrete various cytokines, including interleukin-6 (IL-6), interleukin-11, transforming growth factor (TGF), granulocyte-macrophage colony-stimulating factor, and macrophage colony-stimulating factor (M-CSF). The final signaling molecule in the osteoclastogenesis process is the RANKL, produced by osteoblasts, which binds to its receptor RANK on osteoclasts [29,30].

Osteoprotegerin (OPG), secreted by cells of the stromal osteoblast lineage, acts as a natural antagonist to RANKL [31]. It has been found that estrogen increases the synthesis of OPG [31]. RANKL, identified as the uncoupling factor secreted by osteoblasts, and other factors increase the quantity of pre-osteoclasts in the bone marrow, accelerating bone resorption. Estrogen plays a pivotal role by enhancing OPG secretion and reducing M-CSF and RANK, as previously highlighted [29–32]. The simplest form of MHT is the use of estrogen either alone (in the event of a hysterectomy) or in conjunction with a progestogen. Researchers in the WHI used a combination of MPA 2.5 mg/day and CEE 0.625 mg/day. There are now a plethora of options, including oral micronized 17-oestradiol (often at a dose of 1–2 mg/day) and additional progestogens such dydrogesterone, micronized progesterone or norethisterone acetate. There are several dose and delivery strategies, including transdermal and percutaneous formulations [20]. Transdermal application of 25–50 pg/day of 17-oestradiol is one such example. Transdermal administration of estrogen, which bypasses the liver’s first-pass metabolism, appears to have a lower risk of venous thromboembolic disease and stroke compared to oral administration [33,34]. While the addition of progestogen in MHT is recommended to reduce the possibility of developing endometrial cancer, it is important to note that the benefit–risk profiles of mimic estrogen MHT and anti-estrogen MHT differ significantly. Additionally, tissue selective estrogen complex (including CEE and bazedoxifene) and tibolone (a synthetic steroid with mixed estrogenic, androgenic, and progestogenic action) [35] may be used to alleviate the symptoms of menopause. However, their use and availability vary widely across countries. As a result of these challenges, it is crucial to assess each MHT formulation independently and personalize treatment as much as possible within the bounds of the available information. Additional information on dosage and administration methods may be found in Rozenberg et al. [20,10].

## 3 Role of MHT in disease prevention

Weight gain is not directly caused by menopause; however, low estrogen levels can promote a shift in fat distribution from the hips and thighs to the abdomen and torso. Abdominal obesity increases the likelihood of chronic inflammation, which in turn elevates the risk of developing diseases such as heart disease, diabetes, and certain forms of cancer [36].

### 3.1 Cardiovascular diseases (CVD) risk

**Figure 3:**
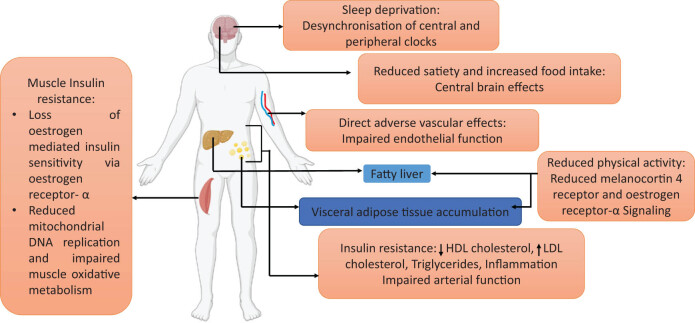
There are several ways in which estrogen decline during menopause might have a negative impact on cardiometabolic health. The decrease in estrogen that occurs during menopause is thought to have direct impacts on the brain, including the potential to lessen satiety and, as a result, increase food intake; it also causes sleep disturbances and lessens muscle’s sensitivity to insulin. In addition, the melanocortin 4 receptor and the estrogen receptor- in the brain are responsible for estrogen’s ability to boost physical activity. This function may be lost after menopause. These alterations, taken together, are a contributor to the rise in visceral adiposity that is associated with menopause, as well as the risk of fatty liver disease. These, in turn, contribute to insulin resistance, which in turn leads to type 2 diabetes mellitus, a higher atherogenic lipid profile (lower HDL cholesterol, increased LDL cholesterol, and triglycerides), and systemic inflammation. Estrogen deficiency has been shown to directly affect arterial endothelial function, which, when combined with a lower lipid profile, raises the risk of CVD [20].

Figure 3 illustrates the various mechanisms by which a deficient supply of estrogen elevates the risk of CVD. CVD encompasses disorders of the heart and blood vessels, including common types such as coronary artery disease, strokes, and hypertension. Risk factors, including high blood pressure, smoking, and obesity, contribute to their onset. Insufficient estrogen levels can result in adverse lipid changes, such as increased levels of triglycerides and LDL cholesterol, along with decreased levels of HDL cholesterol. Additionally, low estrogen can activate the renin-angiotensin system and contribute to a decline in arterial endothelial function.

Furthermore, estrogen insufficiency is associated with risk factors for insulin resistance and type 2 diabetes. These factors include impaired glucose clearance in muscles, increased food intake, and decreased physical activity, as reported [20,37,38]. Animal studies have demonstrated that sleep deprivation during menopause exacerbates insulin resistance and leads to an accumulation of visceral adipose tissue [20,38].

MHT based on estrogen has been found beneficial in various ways. These benefits encompass reduced accumulation of central adipose tissue, improved endothelial function, and lower levels of total and LDL cholesterol. They also include increased levels of HDL cholesterol and triglycerides. However, oral estrogen formulations were associated with a threefold increase in the incidence of venous thromboembolic events [39–49]. Since transdermal estradiol is not subject to first-pass metabolism in the liver, it does not elevate the risk of venous thromboembolic disease or stroke [33,34]. Treatment with estrogen also reduces the risk of developing type 2 diabetes in women without a family history of the disease [20,40]. Despite evidence that estrogen directly improves glucose clearance in muscle and decreases pancreatic β-cell endoplasmic reticulum stress by reducing the buildup of misfolded proinsulin, women might not experience these benefits until later in postmenopause.

Similar to statins, the effects of MHT on the development of atherosclerosis and CVD are believed to be time-dependent [41–43]. Observational studies on symptomatic menopausal women have shown that up to 10 years of MHT usage can have a significant beneficial effect on CVD risk. Thus, for bone protection and fracture prevention, MHT should be primarily considered for treating menopausal symptoms in newly postmenopausal women with a low baseline risk of CVD, cerebrovascular disease, thromboembolic disease, and breast cancer. However, randomized controlled trials that involved asymptomatic women more than 10 years postmenopause found no benefit or potential harm, leading to skepticism regarding the primary prevention of CVD with MHT in a meta-analysis. Nonetheless, according to [20,44], MHT use was associated with a decreased risk of coronary heart disease and overall mortality among women who are less than 10 years past their last menstrual period. For postmenopausal women less than 6 years after their last menstrual cycle, transdermal MHT might offer cardiovascular benefits without elevating the risk of venous thromboembolic events. However, current guidelines do not recommend its use for primary or secondary prevention. Therefore, MHT is not presently advised for the prevention of CVD [20,43].

### 3.2 Other complications

Except for premenopausal women undergoing surgical menopause, menopause is typically not linked with memory loss. Nevertheless, evidence from longitudinal cohort studies suggests that many women undergo a decline in cognitive function after menopause, with memory experiencing the most noticeable drop. Beyond vasomotor symptoms (VMS), sleep disturbances, mood fluctuations, and anxiety, changes in sex steroid hormone levels play a crucial role in memory function during the menopausal transition. Although the role of estrogen in cognitive health is biologically conceivable, there are conflicting results from observational studies and randomized controlled trials. These findings can be both beneficial and detrimental.

Two WHI studies presented evidence of an age-related effect. In WHI substudies, MHT use showed neither harm nor benefit for women who started treatment between the ages of 50 and 55. However, it was associated with negative cognitive effects in women aged 65 and older [44,45]. Two subsequent clinical studies found no positive impact of MHT on cognition [44,45]. Women within 2 years of menopause taking oral hormones reported improved mood and anxiety compared to those using transdermal hormones. Despite the potential of estrogen medication to maintain cognitive function post-surgical menopause, MHT should not be used to enhance cognitive performance in postmenopausal women. Yet, short-term MHT has shown promise in addressing menopausal depression [46,47].

Globally, as many as 12% of women experience early ovarian insufficiency (pre-40 ovarian failure), and up to 4% undergo menopause before age 45. Women with ovaries that cease to function before age 45 face heightened risks of developing type 2 diabetes, early CVD, and premature death, especially if they gain weight and have dyslipidemia [48,49]. According to two meta-analyses [48,49] and most observational studies, oral MHT users possess a reduced risk of developing colorectal cancer. The WHI trials indicated that women who took both MPA and oral conjugated estrogen had a lower risk of colorectal cancer than those taking conjugated estrogen alone [20]. Still, using MHT primarily to prevent colorectal cancer is not recommended. The relative safety of different formulations requires more extensive research [50].

## 4 Effectiveness of MHT in the prevention of fractures

Initial intervention studies were conducted on small groups of women using single or dual-photon absorptiometry to showcase the preservation of bone mass by estrogen, either alone or in conjunction with progestogen [24]. Additionally, US-based studies indicated that the treatment influenced bone histomorphometric parameters [24,51]. The Postmenopausal Estrogen or Progestin Intervention study [12] was the pioneering randomized trial to illustrate that MHT preserves BMD postmenopause using dual-energy X-ray absorptiometry. The WHI determined that MHT reduced the occurrence of hip fractures and other osteoporosis-related fractures in a non-selected sample with low BMD. Over an average follow-up of 5.2 years, a randomized trial with around 16,000 women aged 50–79 years – all of whom had an intact uterus at baseline – revealed a 34% reduction in hip fracture incidence for those who received CEE + MPA compared to a matching control group. Beyond the drop in hip fracture incidence, the WHI study also noted a decline in clinical vertebral fractures and other osteoporotic fractures in the CEE + MPA group in contrast to the placebo [52]. Furthermore, after an added 5.6 years of therapy, the CEE + MPA group maintained a statistically significant reduction in fracture risk. The MHT’s broad efficacy for fracture prevention was underscored by the observation that this risk reduction was uninfluenced by factors such as age, body mass index, smoking habits, personal or family history of falls or fractures, total calcium intake, or prior hormone therapy usage [52]. After 3 years of treatment, the active group experienced a 3.7% increase in total hip BMD, contrasting with a mere 0.14% in the control group [10,53].

The bone-protective effect of MHT must be evaluated when deciding its inclusion in osteoporosis therapy. Women (*n* = 347) from four placebo-controlled MHT trials were followed up 5, 11, and 15 years after completing treatment [54]. While bone loss rates reverted to typical postmenopausal levels after ceasing MHT, the BMD of MHT-treated women remained higher than that of placebo-treated women for several years. Additionally, those previously treated with MHT had a reduced frequency of all osteoporotic fractures compared to the placebo group [54].

However, other research, both mechanistic and population-based, has challenged the alleged preventative benefits of MHT for bone health. Postmenopausal women in the US National Osteoporosis Risk Assessment study, who had discontinued MHT within the past 5 years, had a similar hip fracture risk as those who had never been on MHT [55]. Another study using data from the Kaiser Permanente health management organization in Southern California monitored 80,955 postmenopausal women who had been on MHT for approximately 6.5 years [56]. Compared to continuous MHT users, those who abruptly stopped had a 55% increased risk of hip fracture within the following 2 years. Observational studies, in contrast to randomized trials, tend to be more susceptible to bias and confounding. Three years post-intervention, the WHI trial detected no lasting benefit from MHT use [57]. Yet, during both the intervention and post-intervention periods, the hip fracture rates were lower in the intervention group. Women assigned to either MHT or placebo, then observed for 5 years post-intervention, showed no signs of decreased fracture risk among former MHT users compared to those who had taken a placebo. While the CEE + MPA trial did not observe a significant reduction in overall fractures among past MHT users, the CEE-alone study (focused on women who had undergone hysterectomy before the study) did find some evidence of a lasting effect on total fractures [58–61].

In the WHI trial, 16,089 women were randomly assigned to receive MHT or a placebo and either calcium + vitamin D supplementation or a placebo. This was to ascertain the effectiveness of combining these two interventions to reduce hip fractures. The combination of MHT and calcium + vitamin D supplements proved more effective in preventing hip fractures compared to either treatment alone. A notable reduction in hip fractures was observed among women who received the calcium and vitamin D supplements, as opposed to those given a placebo or hormone treatment alone. However, the combination therapy appeared to accelerate fracture healing [60], but did not influence variations in BMD in the hips and spine.

The bone-protective benefits observed in the WHI trial were supported by a meta-analysis of 28 studies encompassing 33,426 participants and 2,516 fracture cases. The hazard ratio for all fractures (MHT vs placebo) was found to be statistically significant. Zhu et al. [62] reported that the magnitude of effect for hip and spinal fractures was similar. While studies on lower dosages and transdermal applications have shown positive effects on BMD, definitive fracture outcomes remain under-researched [25]. These dosages include oral conjugated estrogens at 0.3 mg/day, micronized 17-estradiol at 0.25 mg/day, and transdermal estradiol at 14 g/day. Estrogen therapy aims to mimic the functions of natural estrogens, namely oestradiol, oestrone, and oestriol. The most frequently prescribed forms of estrogen are available in oral formulations (Table 1) [20,63,64].

**Table 1:** MHT and standard dose per day

	Compound	Dose per day
estrogens	CEEs	0.625 mg
	Micronized 17β-oestradiol	2.0 mg
	Oestradiol valerate	2.0 mg
	Piperazine oestrone sulfate (oestropipate)	0.625 mg
	Transdermal 17β-oestradiol	50 μg
	Subcutaneous implant oestradiol	50 mg
	Oestradiol gel	1 mg
	Oestradiol hemihydrate gel	1.5–2.25 mg
Progestogens	Micronized progesterone	100 mg
	Dydrogesterone	5–10 mg
	Drospirenone	1–2 mg
	Norethisterone acetate	1.0 mg
	MPA	2.5–5 mg
	Levonorgestrel intrauterine device	20 μg levonorgestrel every 24 h initially
Other	Tibolone	2.5 mg
	Conjugated estrogens plus bazedoxifene	0.45 mg plus 20 mg
	Drospirenone	4 mg for 24–28 days for perimenopausal cycle control and contraception

## 5 Incorporating MHT into routine maintenance for bone health

### 5.1 Economics of health care provisioning

Researchers have studied the cost-effectiveness of menopausal hormone therapy (MHT) in treating menopausal symptoms and preventing fragility fractures in asymptomatic women at high risk. Zethraeus et al. [64] examined the societal perspective in Sweden for women between the ages of 50 and 60, integrating efficacy and side effect data from the WHI into an individual state transition model. Lekander et al. [65] conducted a parallel analysis for the UK population using a Markov cohort simulation model for women aged 50 and older. Both investigations, which were based on WHI data, confirmed the cost-effectiveness of MHT [65]. It was shown that cost-effectiveness was largely influenced by the severity of menopausal symptoms, with MHT being deemed cost-effective even for those with moderate symptoms [64].

The studies encompassed women from Sweden, the United States, and the United Kingdom who were at high risk but did not have menopausal symptoms. In the state transition model, fracture, cancer, and CVD were all considered terminal conditions. The evidence for cost-effectiveness was less compelling for women who had never sustained a uterine fracture. However, the data suggested that MHT was more cost-effective than no treatment for those who had undergone a hysterectomy. The pivotal determinant in evaluating cost-effectiveness was the fracture risk. Economically, women who had experienced a hysterectomy and had no prior vertebral fracture seemed to benefit from MHT. Only women with a pristine history of vertebral fracture and an intact uterus were advised against MHT. These results support the use of MHT for alleviating menopausal symptoms but raise questions about its role in fracture prevention, especially for those at a lower risk. The analyses did not account for the duration since menopause, a factor generally believed to enhance cost-effectiveness.

### 5.2 Recommendation and guidelines for MHT

Several international guidelines endorse MHT as a potential first-line therapy to maintain bone health in women just entering menopause. EMAS members concur that the “administration of systemic MHT possesses a favorable risk–benefit profile for women under the age of 60 years or within 10 years postmenopause for both menopausal symptoms and osteoporosis” [66]. The North American Menopause Society asserts that the benefit–risk ratio is optimal for treating prominent VMS and for women under 60, or within 10 years of menopause onset, especially those at high risk for bone loss or fracture and with no contraindications. Recommendations from the International Menopause Society (IMS) [67] mirror this stance. While the American Association of Clinical Endocrinologists highlights that “MHT should be administered at the lowest dose and shortest duration needed to manage menopausal symptoms,” they also stress its use for osteoporosis prevention and treatment within an overall benefit-versus-risk assessment for each patient [68]. The US Endocrine Society notes the lack of consensus regarding MHT’s efficacy in preventing CVD, breast cancer, or dementia but acknowledges its uncertain benefit in fracture prevention [69]. The American College of Obstetricians and Gynecologists emphasizes treating menopausal symptoms, advocating for the “lowest dosage, shortest duration” approach. A Global Consensus Statement from 2016 states: “MHT can commence in postmenopausal women at fracture or osteoporosis risk before age 60 or within 10 years postmenopause.” This sentiment was backed by prominent organizations like the Endocrine Society, the European Menopause Society, the International Osteoporosis Foundation (IOF), the Federation of Latin American Menopause Societies, and the IMS dose [70], the optimal benefits of MHT might be reaped at the lowest feasible dose. The UK’s National Institute for Health and Care Excellence (NICE) champions MHT for postmenopausal symptoms, noting the subdued risks of CVD with estrogen-only MHT and negligible or slightly elevated coronary heart disease risks with combined MHT for those below 60. When devising a treatment strategy, factors like baseline cardiovascular risk, breast cancer status, and history of thromboembolic disease should be considered [10]. The US Preventive Services Taskforce Report stands as a credible source for evidence-based guidance in clinical preventive services, offering a window into the most recent trends in US preventive healthcare, crucial for MHT decisions. With healthcare’s ongoing evolution, such insights are invaluable. The influence of NICE guidelines in the UK and their alignment with European healthcare perspectives provides a broader international context. Their continual updates, capturing the latest in medical science, render them an essential tool for clinicians and patients navigating MHT choices in Europe and beyond.

### 5.3 Therapeutic methodology of MHT

**Figure 4:**
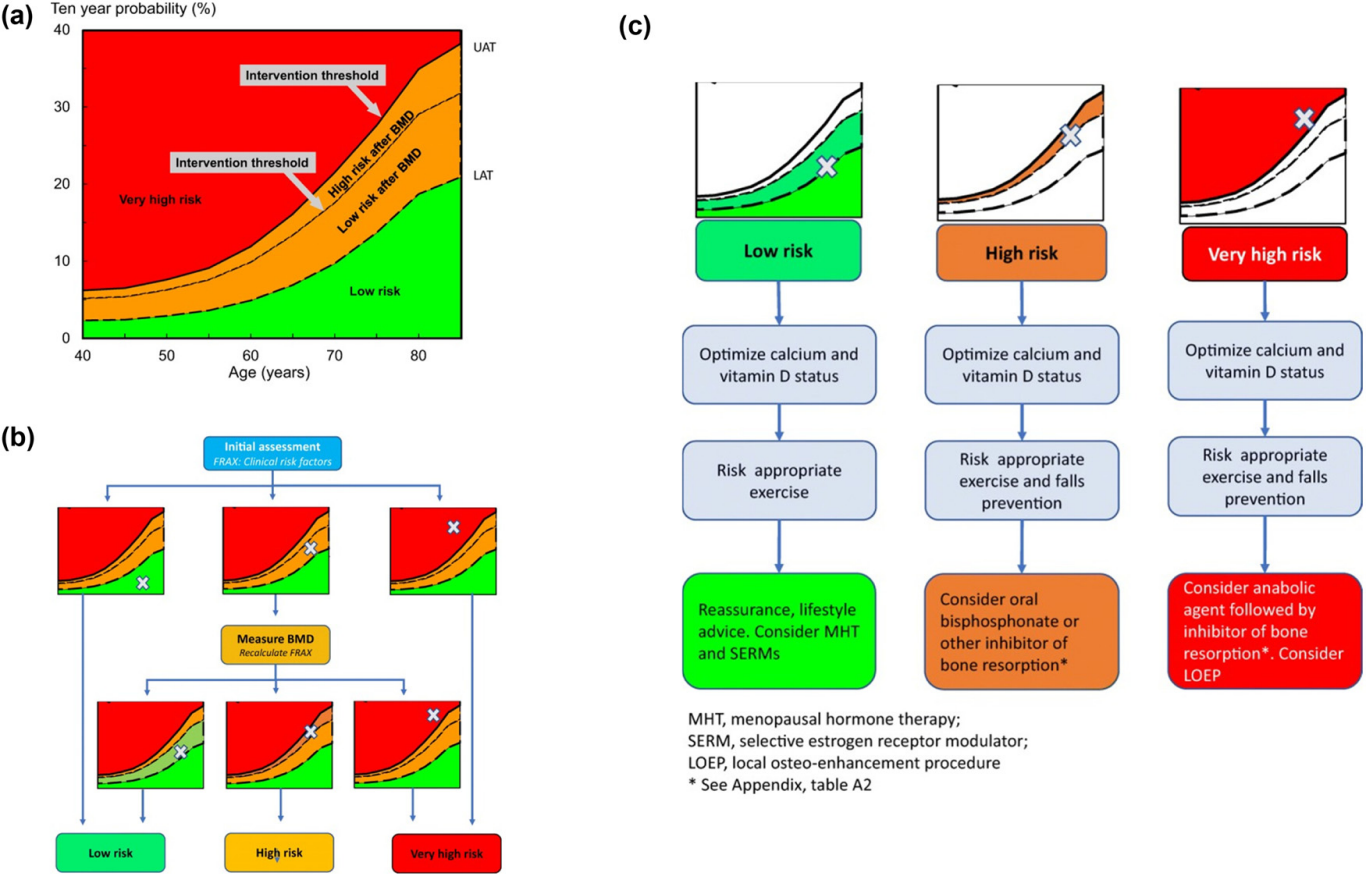
(a and b) An infographic that provides an overview of the classification of fracture risk based on the FRAX major osteoporotic fracture probability in postmenopausal women. The FRAX model is used for the first risk assessment, which only considers clinical risk variables. When the FRAX probability is in the red zone, this indicates a very high risk, and suggests that an initial course of anabolic medication followed by antiresorptive therapy would be warranted. If the FRAX probability is in the green zone, this indicates a low risk, and the patient should be counseled on how to improve their lifestyle, as well as their consumption of calcium and vitamin D, and the therapy of menopausal hormones. If the FRAX probability is in the intermediate (orange) zone, then a BMD examination and recalculation of the FRAX probability should be performed, including taking into account the femoral neck BMD. After recalculation, the risk may fall into the red zone (very high risk), the orange zone (high risk, which indicates starting antiresorptive treatment), or the green zone (low risk, either in the original green zone or in the original orange zone but below the intervention threshold). The red zone represents a very high risk, while the orange zone and green zone both advise initial antiresorptive therapy. Take note that individuals who have had a previous fracture due to fragility are classified as having at least a high risk, and depending on the FRAX likelihood, maybe even a very high risk. (c). Different treatment options are based on the classification of the patient’s risk of fracture. Regarding the various therapeutic approaches (such as bone resorption inhibitors, anabolic medicines, etc.) [71].

The diagnosis of osteoporosis is now feasible thanks to well-established criteria and advanced technology. The disease can also be effectively addressed using a range of established methods. The IOF, the European Society for Clinical and Economic Aspects of Osteoporosis, Osteoarthritis, and Musculoskeletal Diseases, and the European Guidance for Assessment and Treatment of Postmenopausal Osteoporosis (2019) have recently released comprehensive guidelines for categorizing and evaluating individuals at low, high, and very high risk of osteoporotic fractures. Given the diverse pharmaceutical options available, including calcium and vitamin D supplements, bisphosphonates, and anabolic therapy, a risk-based management strategy is crucial. Although the FRAX^®^ algorithm [71] might indicate a reduced risk of fragility fracture over the next decade for postmenopausal women, these women may still face a significantly higher residual lifetime risk (Figure 4). Consequently, MHT might be considered a preventive measure in the initial phase of a broader treatment strategy, especially in situations where a targeted bone therapy, like bisphosphonates, might not be appropriate. The question then arises: should MHT be recommended primarily for the preservation of bone health and fracture prevention, or should the reduction in fracture risk be viewed as a secondary benefit of a medication primarily used to treat menopausal symptoms?

MHT appears beneficial in preventing fractures in individuals with low BMD, regardless of concurrent progestogen use. However, it remains uncertain if the benefits will persist once the medication is discontinued. Various factors, including age, duration of use, dosage, and administration method, might influence the risks and benefits associated with MHT. The KEEPS trial suggested that the potential cardiovascular benefits of unopposed estrogen, initiated within 10 years postmenopause, outweigh those of opposed estrogen. However, there are potential adverse outcomes, including deep vein thrombosis, stroke, and gallbladder disease. The risk of breast cancer has also been highlighted as a potential side effect of combined MHT in the study conducted by Rozenberg et al. [10].

In assessing the potential risks associated with MHT, both the absolute risks of side effects in the menopausal population and the benefit–risk balance of other osteoporosis treatments must be considered. In the United Kingdom, a 50-year-old woman with no known risk factors for CVD has a 2% probability of developing the disease over the next decade. According to the WHI, women with an intact uterus treated with combined MHT face a 34% increased risk of CHD. However, this increase might not be clinically significant (final absolute risk = 2.68%) [8]. Postmenopausal women with modest cardiovascular risk might benefit from a 5-year course of MHT starting at menopause, but the long-term effects remain uncertain. Besides rare yet severe side effects like osteonecrosis of the jaw and atypical femoral fractures, intravenous administration of bisphosphonates and denosumab can lead to symptomatic hypocalcaemia [2,3,72]. Due to the heightened risk of myocardial infarction, the European Medicines Agency imposed restrictions on strontium ranelate’s license. Though the drug was voluntarily withdrawn from the market, it has since returned as a generic. Conversely, romosozumab has been associated with an increased risk of cardiovascular adverse events. Exposure to strontium ranelate has been demonstrated to elevate the risk of DVT [23,72].

More broadly, the challenges and risks of improperly administering oral bisphosphonates should be considered. Therefore, a comprehensive evaluation of MHT’s pros and cons is essential. MHT can mitigate the risk of bone loss and fractures and is also effective in alleviating menopausal symptoms. While the benefit–risk ratio of using MHT primarily for fracture prevention is less established, further exploration of MHT within the framework of a long-term osteoporosis treatment strategy is warranted. Nonetheless, MHT should be viewed as a first-line treatment for women who have recently entered menopause, especially if the primary therapeutic goal is preserving skeletal health and they have a low risk of CVD, thromboembolic disease, and breast cancer. This article does not delve into the clinical assessment of menopausal symptoms or the specific management of these symptoms, including potential risks. All the international and national entities whose recommendations we have referenced provide comprehensive guidance on managing menopause.

## 6 Limitation of MHT

MHT, while beneficial for many individuals, comes with several limitations. First, it is associated with an increased risk of serious health issues, including breast cancer, dementia, stroke, and CVD, particularly with prolonged use [73–83]. This necessitates careful consideration and ongoing monitoring during the treatment process. Second, MHT might not be suitable for all individuals; those with a history of certain types of cancers, blood clots, or liver disease are generally advised against it. Moreover, it can cause side effects such as nausea, headaches, and vaginal bleeding, which can significantly affect the quality of life. The therapeutic regimen of MHT can be complex and may require trial and error to find the most suitable hormone combination and dosage for an individual, which can be time-consuming and potentially frustrating. Furthermore, the beneficial effects of MHT often reverse once the therapy is halted, which can lead to the recurrence of menopausal symptoms. Lastly, there is also the challenge of adherence to therapy (Collaborative Group on Hormonal Factors in Breast Cancer, 2019). The necessity for continuous use and potential side effects can discourage individuals from sticking to the therapy in the long run. It is essential to have a thorough discussion with healthcare providers to understand the potential risks and benefits fully, and to ensure informed decision-making regarding the therapy [75] (Collaborative Group on Hormonal Factors in Breast Cancer, 2019). This underscores the necessity for individualized treatment approaches and the active involvement of patients in managing their health choices.

## 7 Conclusion

Research on MHT is complex, as observational studies and randomized trials have yielded inconsistent results. While subsequent studies have largely exonerated MHT, lingering controversy after the initial WHI findings resulted in a sharp decline in its usage. Postmenopausal women under 60 looking to protect their bone health might opt to initiate MHT around the onset of menopause or shortly thereafter, rather than waiting until they need specific bone-active medications. This decision is informed by MHT’s proven efficacy in alleviating menopausal symptoms. In making this choice, it is crucial to weigh not just the risk of breast cancer but also the potential for CVD, stroke, pulmonary embolism, and bone fracture. This evaluation should encompass the entirety of the patient’s menopausal experience and include a comprehensive discussion of benefits and risks. The outcome of hysterectomy can be influenced by various factors, such as the type, dosage, and administration method of estrogen and progestogen, and the potential presence of estrogen resistance. It is paramount to employ treatments proven to reduce fracture risk and to tailor therapies according to each patient’s unique benefit–risk profile, aiming to prevent fractures and maintain bone integrity. Our research indicates that postmenopausal women with a low risk of CVD and breast cancer may derive the most benefit from MHT for treating menopausal symptoms, with the added advantages of ensuring bone protection and reducing fracture risk.
